# Hospital accreditation in Mexico fails to improve the quality of healthcare: lessons from an impact evaluation

**DOI:** 10.3389/fpubh.2024.1386667

**Published:** 2024-06-18

**Authors:** Juan Pablo Gutiérrez, Miguel Angel Rodriguez, Pilar Torres-Pereda, Hortensia Reyes-Morales

**Affiliations:** ^1^Center for Policy, Population and Health Research, School of Medicine, National Autonomous University of Mexico, Mexico City, Mexico; ^2^Independent Researcher, Mexico City, Mexico; ^3^Center for Health Systems Research, National Institute of Public Health, Cuernavaca, Mexico

**Keywords:** hospital accreditation, quality of healthcare, effective access, Mexico, impact evaluation

## Abstract

Healthcare quality in low- and middle-income countries poses a significant challenge, contributing to heightened mortality rates from treatable conditions. The accreditation of health facilities was part of the former health reform in Mexico, proposed as a mechanism to enhance healthcare quality. This study assesses the performance of hospital accreditation in Mexico, utilizing indicators of effectiveness, efficiency, and safety. Employing a longitudinal approach with controlled interrupted time series analysis (C-ITSA) and fixed effects panel analysis, administrative data from general hospitals in Mexico is scrutinized. Results reveal that hospital accreditation in Mexico fails to enhance healthcare quality and, disconcertingly, indicates deteriorating performance associated with increased hospital mortality. Amidst underfunded health services, the implemented accreditation model proves inadequately designed to uplift care quality. A fundamental redesign of the public hospital accreditation model is imperative, emphasizing incentives for structural enhancement and standardized processes. Addressing the critical challenge of improving care quality is urgent for Mexico’s healthcare system, necessitating swift action to achieve effective access as a benchmark for universal healthcare coverage.

## Introduction

The quest to ensure adequate health services for all is a complicated and non-linear process worldwide. Structural deficiencies that result in poor healthcare quality create barriers to improve health in low- and middle-income countries, that are fighting to decrease mortality for conditions that should be effectively treated by health services as the required knowledge and technologies are available (1). In the current global effort to achieve Universal Health Coverage (UHC), ensuring service quality is imperative for increased access to yield better health outcomes, known as effective access (2, 3).

In this quest to improve healthcare quality, a multifactorial challenge that goes from the structural conditions of services to the effective implementation of standardized processes, political support and commitment are needed (1). In Mexico, an upper-middle income country, structural and process-related challenges exist in both ambulatory and hospital public health services, with the lowest quality services disproportionately impacting vulnerable populations (4–7).

To meet this challenge, the Mexican health care system have designed and implemented diverse mechanism as part of the process of health reforms in the past 20 years. With the preceding reform implemented back in 2003, the country introduced a public health insurance (Seguro Popular) as a part of a so-called Social Protection for Health System, that included a formal process to increase the quality of health services.

The reform in 2003 explicitly signaled the intention to increase the quality of health services, and a process of accreditation was introduced to that end, defined as a mechanism to ensure compliment with a set of minimum requirements to provide health care (8). Thus, facilities that were accredited were expected to provide care for individuals affiliated to the Seguro Popular, so related accreditation to resources (the Seguro Popular resources), as only accredited facilities were allowed to both provide care to those in the Seguro Popular and receive resources from the Seguro Popular (9).

Until 2019 (the reform at the end of 2019 eliminated the Seguro Popular), the accreditation process was described as a mechanism to guarantee that health facilities have the structural capacity, quality and security to provided health services.

Accreditation of health services has been defined as mechanism to continuously improve health services with a self-assessment and peer-review evaluation of performance based on pre-defined standards (10, 11), has been suggested as a potential solution for improving healthcare quality, although evidence for its effectiveness is limited (12–14), and even recent systematic reviews provided contradictory results, with one suggesting that there is evidence that accreditation could contribute to improve performance (15) and the other indicating a lack of effect and proposing that this could be related to the complexity of the process (12). In Mexico, the accreditation process has been proposed as an hybrid of accreditation and licensing of services (10). The Mexican model involved self-assessment at the facility level and a peer-review with evaluators designed at the federal Ministry of Health (MoH), and the outcome (the accreditation) was also an approval to provide care for those affiliated to the Seguro Popular, that is, a type of licensing (8).

The accreditation of hospitals in Mexico was regulated by the Directorate General of Quality and Education in Health (DGCES) within the Ministry of Health (8). Until the end of 2019, the accreditation process was linked to reimbursement of expenses and access to funds for providing care to individuals covered by the Seguro Popular (9). Because accredited hospitals were eligible for the Seguro Popular funds, that potentially created an incentive for state-level Ministries of Health and state governments to have their hospitals accredited (16). However, it is important to note that funds were transferred to the state rather than directly to the hospitals, and accreditation did not guarantee additional funds for the hospital. In some cases, anecdotical reports suggested that high-level staff of state-level Ministries of Health attempted to intervene in the accreditation process by seeking accreditation without fulfilling all requirements.

Qualitative studies suggest that some hospitals in Mexico may have met accreditation criteria by using mobile equipment or staff, or by sharing equipment or staff among multiple hospitals (17).

Given that accreditation aims to ensure healthcare provision with minimum standards and quality, it is reasonable to expect that accreditation would lead to improved health outcomes (11). This study aims to evaluate the performance of hospital accreditation in Mexico in enhancing the quality of healthcare measured by indicators of safety, efficiency and effectiveness.

## Methodology

This is a longitudinal retrospective analysis using administrative records of hospital discharges, hospital structure, and accreditation status and date as data sources. We included for the analysis all public second-level hospitals in the country, that is, all hospitals that were candidates for accreditation during the analytic period.

### Indicators

We used a set of standard quality care indicators to estimate the effectiveness of accreditation on hospitals ‘performance. These indicators, grouped in three dimensions (effectiveness, efficiency, and safety), have been previously used to measure performance of Mexican public hospitals (18, 19) and are based on the framework proposed by Kelley and Hurst (20).

*The effectiveness indicators are the* general in-hospital mortality rate, the in-hospital mortality rate due to acute myocardial infarction (AMI) in individuals 45 years of age or older, and the in-hospital mortality rate due to diabetes mellitus (DM) in individuals 45 years of age or older. AMI and DM in -hospital mortality reflects adequality process of care and are major conditions for the Mexican context. Mortality rates were standardized to consider differences in composition by age groups and sex, between different hospitals, and between different periods.^1^ These set of indicators are expected to have a downward trend after accreditation, as quality improvements should decrease in-hospital mortality.

*The efficiency indicators are* the percentage of hospital occupancy, the average number of days of stay, and the average number of daily surgeries per surgical space. Occupancy is included as it has been suggested that values of approximately 85% are optimal for the operation, while values above 90% compromise the responsiveness of the hospital. The days of stay was included to control average severity.

*The indicator of safety* is the rate of bacteremia, as the ratio of discharges that developed any bacteremia during the stay and the total discharges from the hospital in each period (excluding obstetric and psychiatric discharges). If quality of care improves, the bacteremia rate, *ceteris paribus*, should decrease.

### Data

We used public available data for this analysis, specifically the Automated Subsystem of Hospital Discharges (SAEH), the Equipment, Human Resources and Health Information Infrastructure Subsystem (SINERHIAS), and the Accreditation Reports of the Health Information System (HIS) of the Directorate General of Health Information (DGIS).^2^

The SAEH includes data on discharges for all public hospitals in Mexico, including both those that provided services for individuals with social security and those for the population without social security. The SINERHIAS platform includes data on resources available by facility, including beds, surgical spaces, physicians, and nurses. Data from the accreditation reports provided accreditation status and data of accreditation.

The number of public hospitals increases overtime, so there is also a change in the number of observations in the analysis. The database used integrates data from MoH hospitals and other public hospitals (social security). The data used comprise all public hospitals in the country.

### Analysis

We estimate the effectiveness of accreditation using two complementary approaches: controlled interrupted time series analysis (C-ITSA) and a panel regression model.

The panel regression approach uses each health facility as the unit of analysis with annual observations from 2000 to 2017 using a high-dimensional fixed effects model.

The general estimated model for the panel regression approach is expressed as:


(1) $$y_{it}=\alpha_{i}+\beta_{1}\;.\;\;t_{t}+\beta_{2}\;.\;\;A_{it}+\beta_{3}\;.\;\;T_{it}+\beta_{4}\;.\;\;X_{it}+\varepsilon_{it}$$

where $y_{it}$ is the value of the result indicator in facility i in year t , $A_{it}$ it is a dichotomous variable that takes the value of 1 if the establishment is accredited in period t (and zero otherwise), so $\beta_{2}$ it indicates the change in level at the time of accreditation; $\;T_{it}$ it indicates the period in years after accreditation (it is zero for the years in which the establishment has not been accredited), and $\beta_{3}$ indicates the change in the post-accreditation trend. $X_{it}$ is a vector of characteristics of the establishments that vary over time, $\alpha_{i}$ is the intercept by establishment that captures the fixed individual characteristics that affect the output variable, and $\varepsilon_{it}$ the error term.

The estimation of the panel model is implemented using a linear model with high-dimensional fixed effects, clustered at hospital level (21, 22); fixed effects were also adjusted by group (control/treatment), sector (MoH) and state.

In the C-ITSA, individual hospital information was aggregated to form a unique observation for all accredited hospitals and one unique observation for all non-accredited hospitals.

The C-ITSA assumes that, in the absence of an intervention, the pre-intervention trend will remain constant and have been previously used in the evaluation of healthcare interventions (23–27).

Formally, the effectiveness of the accreditation is estimated by the following model:


(2) $$\begin{array}{l} y_{it}=\alpha +\beta_{1}\;.\;\;t_{t}+\beta_{2}\;.\;\;T_{it}+\beta_{3}\;.\;\;\left(t_{t}\;.\;\;T_{it}\right)+\beta_{4}\;.\;\;z\\ \;\;\;\;\;\;\;\;+\beta_{5}\;.\;\;t_{t}\;.\;\;z+\beta_{6}\;.\;\;T_{it}\;.\;\;z+\beta_{7}\;.\;\;\left(t_{t}\;.\;\;T_{it}\right)\;.\;\;z+\varepsilon_{t} \end{array}$$

where the subscript i identifies the groups ( i=control,treatment ), the subscript (and variable) t is the time indicator that starts at zero, α it is the initial value (at t=0 ) of $y_{it}$ at, $\;T_{it}$ it is a dichotomous variable that identifies whether the group has accreditation in the period t and, finally, z it is a dichotomous variable that identifies the groups (treatment = 1 and control = 0) so it does not depend on t .

The null hypothesis tested in this model is that there is no significant difference in the trend and level changes between the treatment and control groups before and after accreditation.


(3) $$H_{0}:\beta_{6}-\beta_{7}=0$$

We estimated the C-ITSA model with a generalized least squares (GLS) method and the Prais-Winsten transformation to control for serial correlation of errors (28) and robust standard errors.

The control group was formed by matching accredited and non-accredited hospitals on pre-accreditation variables such as hospital characteristics, type of establishment, urban/rural stratum, entity, and demand for specific services, percentage of women of childbearing age attended, and percentage of people over 65 and under 5.

All estimations were implemented with Stata 15 (Stata Corp, TX).

## Results

Table 1 presents the characteristics of the hospitals included in the analysis. By 2017, there were a total of 962 public hospitals: 542 were accredited at that time and 420 were not. In total, we included in the analysis 13,062 hospital-year observations were included, with 51.4% corresponding to accredited hospitals. About 90% of accredited hospitals were operated by the Ministry of Health (MoH) –both federal and states.

**Table 1:** Comparison between accredited and nonaccredited hospitals: characteristics of hospitals, population served and outcome indicators.

Hospitals characteristics					Outcome Indicators				
%	Nonaccredited	Accredited			Indicators	Nonaccredited	Accredited		
		Before	After	Global			Before	After	Global
Ministry of Health	46.4	89.0	92.2	69.3	Days of stay	3.1	2.9	2.8	3.0
Observations	6,247	3,128	3,545	12,917	Observations	6,155	3,098	3,528	12,781
Rural	4.2	2.7	3.4	3.7	Daily surgeries / surgical space	1.2	1.4	1.5	1.4
Observations	6,222	3,119	3,519	12,860	Observations	2,270	2,208	3,175	7,653
Annual discharges	4,251	4,017	3,921	4,104	Occupancy rate	58.0	52.2	56.0	55.9
Observations	6,247	3,128	3,542	12,917	Observations	2,614	1,407	2,033	6,054
Periods with data	11.4	15.4	14.3	13.2	Nurses / contact physician rate	1.8	1.6	1.7	1.7
Observations	6,247	3,128	3,542	12,917	Observations	4,495	2,563	3,497	10,555
Short stays	3.2	2.6	2.6	2.9	General mortality rate (all public sector hospitals)^∑^	8.0	12.0	10.5	10.2
Observations	6,247	3,128	3,542	12,917	Observations	2,897	2,788	3,261	8,946
Psychiatric discharges	6.1	4.4	1.8	4.5	General mortality rate (only MoH hospitals)^β^	12.1	14.2	13.2	12.9
Observations	6,247	3,128	3,542	12,917	Observations	6,247	3,128	3,542	12,917
Obstetric discharges	31.0	48.1	47.0	39.5	Rate of bacteremia^∑^	0.4	0.6	0.3	0.4
Observations	6,247	3,128	3,542	12,917	Observations	2,900	2,795	3,263	8,958
Women on fertile age	44.4	59.9	58.9	51.2	Rate of bacteremia rate (main condition)	1.5	1.3	1.5	1.4
Observations	6,247	3,128	3,542	12,917	Observations	6,237	3,120	3,535	12,892
Individuals 65 years or older	15.2	7.6	9.1	11.7	AMI mortality rate (all public sector hospitals)^∑^	9.0	11.3	12.5	11.2
Observations	6,247	3,128	3,542	12,917	Observations	1,247	1,589	1,964	4,800
individuals 5 years or younger	7.3	9.2	8.8	8.2	AMI mortality rate (only MoH hospitals)^β^	13.3	11.3	12.6	12.5
Observations	6,247	3,128	3,542	12,917	Observations	2,756	1,811	2,003	6,570
					Type II DM mortality rate (all public sector hospitals)^∑^	3.4	4.8	4.2	4.2
					Observations	2,407	2,510	3,020	7,939
					Type II DM mortality rate (only MoH hospitals)^β^	3.5	4.5	4.2	4.0
					Observations	4,249	2,821	3,075	10,145

^∑^ Includes only hospitals of the Ministry of Health (MoH).

^β^ Includes hospitals throughout the public sector (SP): SS, IMSS, ISSSTE, Pemex, Defense, marine, etc. In the case of the general mortality rate, discharges are not excluded under any criteria; In the case of mortality rates due to AMI and type II DM, discharges by origin of another hospital are not excluded.

### Controlled interrupted time series analysis

The results of the Controlled Interrupted Time Series Analysis (C-ITSA) are reported in Table 2.

**Table 2:** Effect of accreditation on performance indicators (controlled interrupted time series analysis and high-dimensional fixed effects).

Variables	Days of hospital stay	Daily surgeries by surgical space	Occupancy rate	Ration nurses/attending physicians	General in-hospital mortality rate (All public hospitals) ^∑^	General in-hospital mortality rate (only MoH hospitals) ^β^	Rate of bacteremia^∑^	Rate of bacteremia (main dx)	In-hospital AMI mortality rate (All public hospitals) ^∑^	In-hospital AMI mortality rate (only MoH hospitals)^β^	In-hospital Diabetes mortality rate (All public hospitals) ^∑^	In-hospital Diabetes mortality rate (only MoH hospitals)^β^
Controlled Interrupted Time Series Analysis^£^												
Month	0.0	0.0***	1.6***	0.0***	−0.4***	−0.3	0.0	0.1***	0.0	0.2	−0.0	−0.2***
Treatment	0.2	0.1	15.2***	0.1	−0.9	4.2	0.1	0.4	−1.2	2.3	2.8***	2.4**
Slope diff. Preintervention	−0.0	0.0	−1.2***	−0.0*	0.4	0.0	0.0	−0.1*	−0.0	−0.2	−0.1*	−0.0
Level change (control)	0.0	0.1	−2.6**	0.0	0.0	0.2	−0.5***	−0.1	1.2	1.6	−0.4	1.2***
Slope change (control)	−0.0	−0.0***	−1.4***	−0.0***	−0.1	0.1	−0.1**	−0.3***	−0.2	0.1	−0.1	0.1
Differential level change	−0.1	0.0	3.5	0.0	−2.0	−0.1	−0.2	0.3	−1.0	−1.4	1.4***	0.5
Differential slope change	0.0*	−0.0***	0.6	0.0	0.4	0.2	0.0	0.2***	0.3	0.0	0.0	−0.0
Constant	2.9***	1.3***	37.3***	1.5***	13.6***	21.1***	0.9**	0.6	36.7***	39.7***	5.7***	6.5***
R-square	0.869	0.821	0.919	0.735	0.730	0.690	0.562	0.559	0.666	0.797	0.796	0.809
Observations (months)	58	54	56	56	57	58	51	58	49	49	56	56
High-dimensional fixed effects												
Time	0.01	0.03**	1.00***	0.02**	0.07	0.09*	−0.03***	0.01	0.24**	0.18*	0.11*	0.15*
Slope diff. Preintervention	0.00	0.02	−0.35	0.01	−0.51***	−0.41***	0.08***	−0.01	−0.37**	−0.36*	−0.07	−0.1
Level change (treatment)	0.10*	−0.07	1.48	−0.04	0.71	1.28***	−0.71***	0.10	0.15	0.12	0.85*	0.95*
Change of slope (treatment)	0.02	−0.06**	−0.09	−0.01	0.34*	0.21	−0.11***	0.04	0.43**	0.50**	0.01	0
Constant	4.06***	−0.12	46.97***	1.50***	3.24	10.04***	0.17	1.18	−3.75	−7.87	5.45	5.46
R-square	0.804	0.655	0.779	0.711	0.770	0.853	0.126	0.493	0.362	0.479	0.483	0.555
Observations	12,762	7,632	6,003	10,536	8,820	12,762	8,940	12,873	4,735	6,474	7,915	10,114

Statistical significance: *p < 0.05, **p < 0.01, ***p < 0.001.

^£^ The daily average of discharges is included as a control variable.

^Ω^ A linear models with high-dimensional fixed effects were adjusted, with fixed effects nested within cluster at hospital level; fixed effects were also adjusted at group (control/treatment), sector (MoH) and state level. Control variables included in the model are daily average of discharges, percentage of short stays (with respect to total discharges), of psychiatric, obstetric discharges, percentage of women of childbearing age, percentage of older adults (65+) and of under 5 years old Hospitals are weighted by the average annual discharges. Hospitals where weighted using the average daily discharges, so that those hospital with more discharges has bigger weight on the regression.

^∑^ Includes only hospitals of the Ministry of Health (MoH).

^β^ Includes hospitals throughout the public sector (SS, IMSS, ISSSTE, Pemex, Defense, Marine, etc.). In the case of the general mortality rate, discharges are not excluded under any criteria; In the case of mortality rates due to AMI and type II DM, discharges by origin of another hospital are not excluded.

Prior to accreditation, no significant differences in levels or trends were observed between the two groups. However, post-accreditation, significant differences emerged. Rates of diabetes in-hospital mortality were higher at accredited hospitals, with a marginal and negative difference in the slope (indicating that among accredited hospitals, the rate decreases faster compared to non-accredited ones). On the other hand, the ratio of nurses per attending physician has a marginally greater decreasing trend in accredited hospitals, and the occupancy rate is higher in those accredited by 15.2 percentage points, but with a trend 1.2 percentage points lower in accredited hospitals, compared to non-accredited hospitals.

After accreditation, there was an unexpected increase in the rate of in-hospital mortality due to diabetes in accredited hospitals, significantly higher than in non-accredited hospitals, which was significantly higher than that observed in non-accredited hospitals (1.4 death per 1 thousand discharges). However, there were no differences in the trend. Additionally, there is a change in the slope of the bacteraemia rate among accredited hospitals (change in slope 0.2 points higher).

In turn, among accredited hospitals, changes in the slope of magnitudes can be observed to be close to zero for the average number of days of stay and the daily average of surgeries per surgical setting.

### Panel analysis

The findings from the high dimensional fixed effects (HDFE) panel regression model are presented in Table 2, aligning with the results of the Controlled Interrupted Time Series Analysis (C-ITSA).

#### General in-hospital mortality rate

The HDFE model identified a significant increase in mortality post-accreditation, with an increment of 1.28 deaths per 1,000 discharges specifically in Ministry of Health (MoH) hospitals. For the broader set of all public hospitals, while there was no change in the initial level, a significant upward trend of 0.34 deaths per 1,000 discharges was observed.

#### In-hospital AMI mortality rate

For Acute Myocardial Infarction (AMI), there was a noticeable rise in the mortality rate slope, increasing by 0.50 deaths per 1,000 discharges per period in MoH hospitals, and by 0.43 deaths per 1,000 discharges per period across all public hospitals.

#### In-hospital diabetes mortality rate

Post-accreditation, the diabetes mortality rate showed a significant increase in MoH hospitals, with an uptick of 0.95 deaths per 1,000 discharges, and a similar rise of 0.85 deaths per 1,000 discharges across all public facilities.

#### Bacteraemia rates

No significant changes were observed in the rates of bacteraemia as the primary condition. However, when estimated as a secondary condition, there was a notable reduction in both the level (−0.71) and trend (−0.11 points) post-accreditation.

#### Hospital stay duration

Additionally, there was a slight increase in the average hospital stay duration, extending by 0.10 days following accreditation.

## Discussion

In this study, a Controlled Time Series Analysis (C-ITSA) and a panel regression model with high dimensional fixed effects (HDFE) were employed to assess the effectiveness of accreditation on various quality measures in Mexican hospitals. The results from both analyses consistently indicate that the accreditation of hospitals in Mexico has not led to significant positive changes in the effectiveness, efficiency, and safety of health services provision. Moreover, the study suggests potential perverse effects of accreditation, specifically an increase in in-hospital mortality rates.

The reason for the increase in in-hospital mortality is unclear. We hypothesized as one potential explanation that after accreditation, hospitals may receive more severe patients due to an increase in technical capabilities. However, this increase in mortality is not limited to general mortality but also includes mortality from acute myocardial infarction, which already indicates patient severity. Additionally, despite the accreditation status being publicly available, it is not widely promoted to potential users, making it unlikely for patients to select a hospital based on accreditation status alone.

The results indicate marginal changes toward greater efficiency (higher occupancy rate and greater number of surgeries per surgical setting), but without this being reflected in the overall effectiveness and safety of care.

While these results do not align with studies from other countries that have found a potential link between accreditation and improved health outcomes (15, 29–31), it is important to note that the Mexican accreditation system is a hybrid of licensing and accreditation, which affects comparability. However, these results are consistent with other studies that have specifically examined the association between accreditation and health outcomes, which have found no correlation between accreditation and lower hospital mortality (12, 32).

A related study from United Arab Emirates using interrupted time series analysis suggested that performance improved in the pre-accreditation period (during the preparation for accreditation) but some of the gains were lost after accreditation although positive results were maintained at least after 3 years (33).

In the Mexican context, it is noteworthy that while accreditation was established to ensure minimum standards, it was also viewed as a means to secure additional funding at the state level Ministries of Health. Prior analyses in Mexico have highlighted the potential for accreditation to be perceived by facilities and state governments as a means to acquire additional resources, rather than as a tool to enhance the quality and safety of patient care (7).

The potential influence of state governments on the accreditation process highlights the importance of independent regulators that are less susceptible to political pressure. In countries with established experience in measuring quality assurance as the core component of accreditation, the process is conducted by an external evaluator, such as an independent, primarily non-governmental organization. However, in Mexico, accreditation is carried out by the Ministry of Health, which means that accreditation and service provision are performed by the same institution, limiting transparency of the process (34, 35).

The trend of hospital mortality rate due to acute myocardial infarction (AMI) found in this study is consistent with that reported in other studies. A recent report also identified a growing trend in this indicator between 2010 and 2015. The same study noted that the death rate increased in line with an increase in the demand for care for AMI, doubling between 2002 and 2013. This exacerbates the challenge, and there are significant deficiencies in the capacity of hospitals to care for AMI [Secretaría (36)].

It is clear that no changes are indicating greater efficiency in services, but rather the opposite, except for an increase in the percentage of occupancy, although it is still below 85% (considered an adequate minimum).

This analysis does not permit identification of the reasons for the lack of positive results from accreditation, but the outcome may suggest insufficient investment in strengthening the structural capacity of services. This creates apparent inefficiencies as the actual capacity of services does not match the reported capacity when spaces and services are considered outdated or understaffed (5, 7).

A limitation of the analytical approach using interrupted time series is the assumption that trends in indicators would have remained unchanged in the absence of accreditation. This generally implies that there are no other interventions that occurred while each hospital was accredited that could have resulted in changes in indicators. While this assumption may be strict, the fact that the analysis has been conducted on a broad set of indicators with consistent results among them, and the trends have been compared with non-accredited hospitals, allows us to be confident that the results are robust.

Another consideration is that the results are affected by the available data, which is lower for the earlier years in the period for which information is available. To address this limitation, a weighted average of indicators in accredited establishments was used. Additionally, to ensure that the intervention was adequately identified, the analysis aligns the hospitals in time to when they received accreditation.

As previously stated, providing health services without ensuring adequate levels of quality not only represents a waste of resources but is also not ethical and is not attentive to human rights (1). As emphasized in the literature, accreditation processes must have indicators oriented toward the desired results, namely better outcomes for the population served (37).

## Conclusion

The accreditation model for establishments in Mexico has not resulted in improvements in health outcomes for patients, at least in the case of second-level hospitals. The unexpected increase in in-hospital mortality rates and the absence of significant positive changes in various indicators call for a reassessment of the accreditation process. Consideration should be given to the hybrid nature of the system, financial incentives, and the potential influence of state governments. Additionally, the findings underscore the importance of transparent, independent regulators to ensure the integrity and efficacy of accreditation in promoting quality healthcare.

## Data availability statement

Publicly available datasets were analyzed in this study. This data can be found at: http://sinaiscap.salud.gob.mx:8080/DGIS/.

## Ethics statement

Ethical approval was not required for the study involving humans in accordance with the local legislation and institutional requirements. Written informed consent to participate in this study was not required from the participants or the participants’ legal guardians/next of kin in accordance with the national legislation and the institutional requirements.

## Author contributions

JG: Conceptualization, Formal analysis, Methodology, Supervision, Writing – original draft, Writing – review & editing. MR: Data curation, Formal analysis, Writing – original draft, Writing – review & editing. PT-P: Conceptualization, Writing – review & editing. HR-M: Conceptualization, Writing – review & editing.

## Footnotes

^1^Direct standardization was used. However, to avoid data losses, for those years that a hospital did no reported discharges for the analyzed causes, we assumed a mortality rate equal to zero.

^2^ http://sinaiscap.salud.gob.mx:8080/DGIS/
